# Aerobic exercise and intraocular pressure in normotensive and glaucoma patients

**DOI:** 10.1186/1471-2415-9-6

**Published:** 2009-08-13

**Authors:** Konstantinos Natsis, Irene Asouhidou, George Nousios, Theodosios Chatzibalis, Konstantinos Vlasis, Vasilios Karabatakis

**Affiliations:** 1 Department of Anatomy, Medical School, Aristotle University of Thessaloniki, Greece; 2 2nd Department of Anaesthesiology "G.Papanikolaou" Regional Hospital, Thessaloniki, Greece; 3 Laboratory of Anatomy, Department of Physical Education and Sport Sciences (Serres), Aristotle University of Thessaloniki, Greece; 4 Laboratory of Experimental Ophthalmology, Aristotle University of Thessaloniki, Greece

## Abstract

**Background:**

With the increasing number of people participating in physical aerobic exercise, jogging in particular, we considered that it would be worth knowing if there are should be limits to the exercise with regard to the intraocular pressure (IOP) of the eyes. The purpose of this study is to check IOP in healthy and primary glaucoma patients after aerobic exercise.

**Methods:**

145 individuals were subdivided into seven groups: normotensives who exercised regularly (Group A); normotensives in whose right eye (RE) timolol maleate 0.5% (Group B), latanoprost 0.005% (Group C), or brimonidine tartrate 0.2% (Group D) was instilled; and primary glaucoma patients under monotherapy with β-blockers (Group E), prostaglandin analogues (Group F) or combined antiglaucoma treatment (Group G) instilled in both eyes. The IOP of both eyes was measured before and after exercise.

**Results:**

A statistically significant decrease was found in IOP during jogging. The aerobic exercise reduces the IOP in those eyes where a b-blocker, a prostaglandin analogue or an α-agonist was previously instilled. The IOP is also decreased in glaucoma patients who are already under antiglaucoma treatment.

**Conclusion:**

There is no ocular restriction for simple glaucoma patients in performing aerobic physical activity.

## Background

As an increasing number of people are becoming active in aerobic physical exercise such as jogging and bicycling it would be interesting to identify any limitations or precautions, concerning the effect of exercise on intraocular pressure (IOP). Previous studies have shown a reduction in IOP following certain forms of exercise, ranging from walking to exhausting exercise, in healthy volunteers. According to Qureshi et al all forms of physical exercise (bicycling, walking and jogging) decrease IOP [1]. It is also well known that intraocular pressure decreases after severe exercise to exhaustion [2]. However, the effect of exercise on the IOP for individuals under antiglaucoma medication has not been extensively studied. The present study was designed in order to detect how IOP is affected in athletes, non athletes and glaucoma patients that perform jogging or bicycling with or without instillation of various antiglaucoma eye drugs. The study dealt with this issue using a significant number of individuals (totally 145 individuals) including normotensive individuals and glaucoma patients.

## Methods

One hundred healthy individuals and forty-five primary (open-angle) glaucoma patients were included in the study. This study was approved by the local Ethics Committee of our University and all patients signed an informed consent before the start of the trial. Each subject also underwent a preliminary ophthalmologic examination before participating in the study. The healthy individuals had no history of ocular or systematic diseases and were not under any topical or systemic medication. All glaucoma patients suffered from primary open angle glaucoma (POAG) and were under various antiglaucoma eye drops while four of them suffered from hypertention and were receiving appropriate treatment.

Glaucoma patients and healthy individuals were divided into seven groups (demographic data shown in Table 1):

**Table 1:** Demographics data of participants individuals.

**Group**	**male**	**female**	**Age/Mean age**	**total**
**A **normotensive no medication was instilled	16	9	13–18/15.5	25

**B **normotensive b-blocker in RE	24	16	20–51/35,9	40

**C **Normotensive prostaglandin analogue in RE	18	2	27–55/36,7	20

**D **normotensive a-agonist in RE	10	5	17–38/28,7	15

**E **primary glaucoma under b-blockers	8	7	47–78/62,5	15

**F **primary glaucoma under prostaglandin analogues	7	8	50–75/61	15

**G **primary glaucoma under combination of anti-glaucoma drugs	5	10	50–78/64	15

**Total**				145

RE: right eye

**Group A **consisted of 25 normotensive individuals who exercised regularly. In those subjects just after the completion of 20 minutes of sub-maximal (70%) aerobic exercise (jogging) the IOP on both eyes was measured again. There was no instillation of any drug in their eyes.

**Group B **consisted of 40 normotensive individuals. All the subjects in this group had their right eye instilled with timolol maleate (β-blocker) 0.5%.

**Group C **consisted of 20 normotensive individuals. All the subjects in this group had their right eyes instilled with latanoprost (prostaglandin analogue) 0.005%.

**Group D **consisted of 15 normotensive individuals. All the subjects in this group had their right eyes instilled with brimonidine tartrate (α-agonist) 0.2%.

**Group E **consisted of 15 POAG patients who were under monotherapy with timolol maleate (β-blocker) in both eyes.

**Group F **consisted of 15 POAG patients who were under monotherapy with latanoprost (prostaglandin analogue) in both eyes.

**Group G **consisted of 15 POAG patients. Thirteen patients received a combination of two anti-glaucoma drugs (latanoprost + timolol maleate or timolol maleate + dorzolamide or timolol maleate + brimonidine) and two out of the 15 received a combination of three drugs. (latanoprost + timolol maleate + dorzolamide or timolol maleate + dorzolamide + brimonidine).

The IOP of both eyes was measured before exercise (Goldmann applanation tonometry) and was measured again 2 hours after the instillation of the eye drops. Only in case of Group F the IOP was measured after 12 hours in order to measure the IOP under the influence of both exercise and drug therapy. Within 3 minutes after the second measurement, the individuals were asked to start the exercise on a bicycle ergometer (about 10 minutes of duration at 60–80 Watts, in accordance to the preference of each individual so as to perform a moderate-sub maximal-exercise). The IOP of both eyes was measured again within 5 minutes after the completion of the exercise on a bicycle ergometer. In Groups E, F and G, blood pressure and heart rate were measured 5 minutes before and 5 minutes after the exercise using the Critikon/Dianamap (Vital Signs Monitor 8100T) device. Taking into account the fact that blood pressure and heart rate (HR) tend to increase during exercise, the individuals were instructed to decelerate the exercise when their pulse rates increased over 110/minute. Blood pressure and HR were measured using a non-invasive method (automatic dynameter). Every IOP measurement was performed at least twice and in case of more than a 2 mmHg difference a third measurement was performed, finally taking into account the mean of the two higher values.

All measurements were performed in the morning, from 8:00 to 11:00 a.m. One operator was responsible for installation of the eye drops while the other operator performed the measurements being blinded to the drug therapy of the patient.

As Group A was the control group, we compared the post-exercise IOP reduction between healthy individuals and POAG patients taking into account the right eyes of the healthy individuals of Groups B and C, where we instilled the antiglaucoma eye drops (β-blockers or prostaglandin analogues), and the right eyes of the corresponding glaucoma patients (Groups E and F). We also compared the IOP of the right eyes of healthy individuals in Group A (no drug instillation) with the IOP of the right eyes of glaucoma patients of Groups E and F (monotherapy with a β-blocker or a prostaglandin analogue) before and after exercise. Independent-samples t-tests were used to compare the IOP of these groups of subjects before and after exercise.

Statistical analysis was made using the SPSS Version 11.5 for Windows. According to our calculation a number of at least 15 patients per group were needed in order to achieve a power of 90% with an a-level of 0.05.

## Results

The mean IOP of the right and left eyes for all groups before the instillation of the drops, as well as before and after exercise are summarized in Table 2, 3, 4, 5, 6, 7 and 8. Pair wise comparisons of the means in the three different conditions for the right and left eyes separately, revealed statistically significant differences (p < 0.05). For all groups there was no statistically significant difference (p > 0.05) between the right and left eyes.

**Table 2:** Group A

**IOP**	**RIGHT EYE**	**LEFT EYE**
**Before exercise**	13.36 ± 1.786 mmHg) (range 10–17 mmHg)	13.16 ± 1.540 mmHg (range 10–17 mmHg)

**After exercise**	10.6 ± 2.240 mmHg (range 7–16 mmHg) **p < 0.05**	10.58 ± 2.105 mmHg (range 8–17 mmHg) **p < 0.05**

**Table 3:** Group B

**IOP**	**RIGHT EYE**	**LEFT EYE**
**Before instillation of timolol maleate 0.5%**	15.75 ± 1.46 mmHg (range 12–18 mmHg)	15.62 ± 1.37 mmHg) (range 12–18 mmHg)

**2 h after instillation of timolol maleate 0.5%**	11.92 ± 1.37 mmHg (range 8–14 mmHg)	13.90 ± 1.28 mmHg (range 11–16 mmHg)

**After exercise**	9.80 ± 1.36 mmHg (range 6–12 mmHg) **p < 0.05**	11.65 ± 1.51 mmHg (range 8–15 mmHg) **p < 0.05**

**Table 4:** Group C

**IOP**	**RIGHT EYE**	**LEFT EYE**
**Before instillation of latanoprost 0.005%**	14.08 ± 1.78 mmHg (range 11–18 mmHg)	14.35 ± 2.10 mmHg (range 11–20 mmHg)

**12 h after instillation of latanoprost 0.005%**	11.10 ± 1.79 mmHg (range 7–14 mmHg)	14.25 ± 1.66 mmHg (range 11.5–18 mmHg)

**After exercise**	9.25 ± 1.90 mmHg (range 5–12.5 mmHg) **p < 0.05**	12.13 ± 1.58 mmHg (range 9–15 mmHg) **p < 0.05**

**Table 5:** Group D

**IOP**	**RIGHT EYE**	**LEFT EYE**
**Before instillation of brimonidine tartrate 0.2%**	14.66 ± 2.38 mmHg (range 9–19 mmHg)	14.06 ± 2.08 mmHg (range 9–17 mmHg)

**2 h after instillation of brimonidine tartrate 0.2%**	10.26 ± 2.34 mmHg (range 6–16 mmHg) **p < 0.05**	13.06 ± 2.73 mmHg (range 7–17 mmHg) **p < 0.05**

**After exercise**	7.4 ± 2.02 mmHg (range 5–11 mmHg)	10.4 ± 2.55 mmHg (range 6–15 mmHg)

**mean reduction of IOP after the exercise**	2.86 ± 2.09 mmHg **p < 0.05**	2.66 ± 1.67 mmHg **p < 0.05**

**Table 6:** Group E

**IOP**	**RIGHT EYE**	**LEFT EYE**
**2 h after instillation of b-blocker**	17.13 ± 2.39 mmHg (range 12–20 mmHg) **p < 0.05**	16.27 ± 2.31 mmHg (range 12–19 mmHg) **p < 0.05**

**After exercise**	14.53 ± 2.35 mmHg (range 10–18 mmHg)	13.73 ± 2.31 mmHg (range 10–17 mmHg)

**mean reduction of IOP after the exercise**	2.6 ± 0.81 mmHg **p < 0.05**	2.53 ± 1.11 mmHg **p < 0.05**

**Table 7:** Group F

**IOP**	**RIGHT EYE**	**LEFT EYE**
**12 h after instillation of prostaglandin analogue**	15.93 ± 2.31 mmHg (range 13–19 mmHg)	15.6 ± 2.47 mmHg (range 13–19 mmHg)

**After exercise**	13.60 ± 1.80 mmHg (range 12–16 mmHg)	13.13 ± 2.45 mmHg (range 11–17 mmHg)

**mean reduction of IOP after the exercise**	2.33 ± 0.82 mmHg **p < 0.05**	2.47 ± 0.52 mmHg **p < 0.05**

**Table 8:** Group G

**IOP**	**RIGHT EYE**	**LEFT EYE**
**After instillation of anti-glaucoma drugs**	16.733 ± 2.344 mmHg (range 12–20 mmHg)	18.50 ± 3.041 mmHg (range 14.5–25 mmHg)

**After exercise**	14.267 ± 1.791 mmHg (range 12–17 mmHg) **p < 0.05**	15.667 ± 2.609 mmHg) (range 12–21 mmHg) **p < 0.05**

**mean reduction of IOP after the exercise**	2.467 ± 1.125 mmHg	2.833 ± 1.248 mmHg

An independent-samples t-test was conducted to compare the IOP reduction after exercise of the right eyes of the individuals in Group B with the IOP reduction after exercise of the right eyes of the individuals in Group E. There was a significant difference between IOP reduction in healthy individuals with a β-blocker instillation in their right eye (2.13 ± 0.72) and IOP reduction in glaucoma patients under β-blocker monotherapy (2.6 ± 0.81) after exercise (t = -2.104, p = 0.04). The magnitude of the differences in the means was moderate (eta squared = 0.077).

Then, an independent-samples t-test was conducted to compare the IOP reduction after exercise of the right eyes in Group C with the IOP reduction after exercise of the right eyes in Group F. There was no significant difference in IOP reduction in healthy individuals with a prostaglandin analogue instillation in their right eye (1.85 ± 0.71) and IOP reduction in glaucoma patients under prostaglandin analogue monotherapy (2.33 ± 0.82) after exercise (t = -1.871, p = 0.07). The magnitude of the differences in the means was moderate (eta squared = 0.095).

Finally we conducted an independent-samples t-test in order to compare the IOP reduction after exercise of the right eyes in healthy individuals of Group A (no drug instillation) with the IOP reduction after exercise of the right eyes in glaucoma patients of Groups E and F (monotherapy with a β-blocker or a prostaglandin analogue). There was no significant difference in IOP reduction in athletes (2.92 ± 1.89) and IOP reduction in glaucoma patients under β-blocker or prostaglandin analogue monotherapy (2.47 ± 0.81) after exercise (t = 1.114, p = 0.274). The magnitude of the differences in the means was very small (eta squared = 0.023).

**Figure 1:**
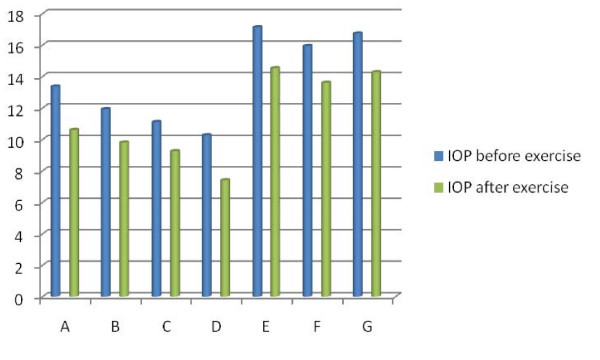
**Graphical demonstration of IOP alterations after exercise in all Groups of individuals**.

In Figure 1 is demonstrated graphically IOP alterations after exercise in all Groups of individuals.

## Discussion

The reduction of IOP after exercise has been the subject of various investigations [3-17]. In normal subjects the intraocular pressure decreases during exercise [18], and its reduction is proportional to the work load [19]. In the present high-powered study we observed that in all groups there was an IOP reduction after aerobic exercise regardless of antiglaucoma eye drop instillation.

Additionally, according to our results, in 40 healthy individuals there was a further reduction of IOP after aerobic exercise, regardless of the instillation of a β-blocker uniocularly. Instillation of the β-blocker does not counterbalance the effect of exercise on IOP. Both eyes of the individuals had lower post-exercise IOP in a similar manner (there was no statistical difference between two eyes). Similarly, glaucoma patients who were under monotherapy with a β-blocker had lower post exercise IOP. We also noticed a significant difference between IOP reduction in healthy individuals having a β-blocker instilled in their right eye (2.13 ± 0.72) and IOP reduction in glaucoma patients under β-blocker monotherapy (2.6 ± 0.81) after exercise (t = -2.104, p = 0.04), although the magnitude of the mean difference was moderate (eta squared = 0.077). Harris et al [5] also reported that the IOP reduced in six individuals with the use of a selective β_1_-blocking drug (betaxolol) and on seven other individuals with a non-selective β-blocker (levobunolol) after physical exercise.

The instillation of prostaglandins also does not prohibit IOP reduction after exercise. As we observed in 20 healthy individuals, in whom a prostaglandin analogue was instilled uniocularly, IOP reduction after the aerobic exercise was almost identical in both eyes. The same was observed in glaucoma patients but there was no significant difference in IOP reduction in healthy individuals with a prostaglandin analogue instillation in their right eye (1.85 ± 0.71) and IOP reduction in glaucoma patients under prostaglandin analogue monotherapy (2.33 ± 0.82) after exercise (t = -1.871, p = 0.07). We may assume that the uveoscleral outflow still increases the aqueous outflow as a response to the aerobic exercise, despite the instillation of prostaglandin eye drops. One may assume that the outflow of aqueous through the trabeculum is increased or the production of aqueous from the ciliary processes is lowered as a result of the aerobic exercise.

Similar assumptions can be made on the other antiglaucoma eye drops we used in the study. The mechanism of IOP reduction is under constant consideration. Martin et al demonstrated that acute dynamic exercise seems to alter IOP through changes in colloid osmotic pressure [10]. A relationship between increased plasma osmolarity and IOP reduction was also suggested by Ashkenazi et al. [3] However, Stewart et al noted that exercise induced greater changes in IOP than oral doses of glycerin for the same change in serum osmolarity [16]. Also, Harris et al suggested that the reduction of IOP correlated with the increase in blood lactate but they did not find any correlation with the plasma osmolarity or the PCO_2_. A previous study by Kielar et al noted that blood lactate and pH changes correlated with intraocular tension changes at anaerobic exercise levels but they did not correlated with intraocular tension changes at aerobic exercise levels [10]. Stewart et al also correlated the effect of exercise on IOP with the nor-epinephrine blood concentration. Lanigan et al reported IOP responses as a result of systemic autonomic stimulations [9]. Orgul and Flammer reported that moderate exercise (6 deep knee bents) with a few seconds of duration can reduce IOP [12]. They correlated this reduction with changes in the heart rate and concluded that IOP reduction was the result of sympathetic activity. In one of our studies, no linear correlations between blood pressure, heart rate and IOP changes were revealed, following aerobic exercise in healthy individuals [7].

It has also been suggested that exercise increases the facility of outflow. The outflow channels of the eye, especially around Schlemm's canal, show fibrinolytic activity [20]. Such fibrinolysis can be postulated to assist in preventing obstruction of the aqueous outflow pathways, and thus aid in the regulation of intraocular pressure. Since exercise increases systemic fibrinolytic activity [21], one can speculate that exercise decreases intraocular pressure by facilitating outflow, although one study revealed no change in facility of outflow when measured immediately following exercise [16].

As for glaucoma patients, regardless of the antiglaucoma medication instilled, they still benefited from the aerobic exercise since they all had a post-exercise reduction of IOP. It is obvious that these patients should be encouraged to perform aerobic exercise. Qureshi [14] also examined 14 individuals (7 normal and 7 glaucoma patients) and noticed that glaucoma patients had a greater IOP reduction and longer duration of post-exercise recovery in comparison to normal individuals. Although the reduction of IOP after exercise lasts for about one hour, this IOP reduction is considered beneficial for the eye. Passo et al [13] in a study of 9 individuals stated that aerobic exercise is associated with a reduction in increased IOP. In this study aerobic exercise was suggested as an effective non-pharmacologic intervention for these individuals. Shapiro et al also proved that the reduction of the IOP in twelve simple glaucoma chronic patients under physical exercise [22]. Our results further confirm that we should encourage glaucoma patients to perform aerobic exercise. Still exercise should be advised with caution for glaucoma patients suffering from pigmentary [6,11], congenital or juvenile glaucoma. [15].

There are certain limitations to our study. There is a significant age difference among the normotensive subjects and glaucoma patients. This is a result of the nature of the disease (primary open angle glaucoma) that presents mainly in elderly people. In any case it has to be pointed out that the mechanism of the IOP reduction of each antiglaucoma drug is always the same regardless of age. Also the mechanism of IOP reduction after prostaglandin analogue requires certain time (1–2 weeks) for the establishment of the potency of the drug. However, even a single instillation of prostaglandin analogues has an IOP lowering effect [23]. All measurements of IOP were performed seven hours after exercise in order to investigate the combination of prostaglandin analogue plus exercise.

## Conclusion

Moderate aerobic exercise reduces the IOP of healthy individuals (athletes or non athletes). Aerobic exercise also reduces the IOP following instillation of a β-blocker, a prostaglandin analogue or an α-agonist. Åxercise reduces the IOP of glaucoma patients already under antiglaucoma treatment. Regular moderate aerobic exercise (walking, jogging, bicycle etc) has been proven beneficial and should be encouraged for glaucoma patients.

## Competing interests

The authors declare that they have no competing interests.

## Authors' contributions

KN, VK, KV, GN and ThC participated in the design of the study and drafted the manuscript. IA and KN edited and revised the final manuscript. All authors have read and approved the final manuscript.

## Pre-publication history

The pre-publication history for this paper can be accessed here:


